# Model instability in predictive exchange rate regressions

**DOI:** 10.1002/for.2620

**Published:** 2019-12-03

**Authors:** Niko Hauzenberger, Florian Huber

**Affiliations:** ^1^ Department of Economics WU Vienna University of Economics and Business Vienna Austria; ^2^ Salzburg Centre of European Union Studies (SCEUS) Paris Lodron University of Salzburg Salzburg Austria

**Keywords:** empirical exchange rate models, exchange rate fundamentals, Markov switching

## Abstract

In this paper we aim to improve existing empirical exchange rate models by accounting for uncertainty with respect to the underlying structural representation. Within a flexible Bayesian framework, our modeling approach assumes that different regimes are characterized by commonly used structural exchange rate models, with transitions across regimes being driven by a Markov process. We assume a time‐varying transition probability matrix with transition probabilities depending on a measure of the monetary policy stance of the central bank at home and in the USA. We apply this model to a set of eight exchange rates against the US dollar. In a forecasting exercise, we show that model evidence varies over time, and a model approach that takes this empirical evidence seriously yields more accurate density forecasts for most currency pairs considered.

## 1 INTRODUCTION

Since the end of the Bretton Woods system in 1971, economists have been confronted with the challenging issue of designing empirical models of bilateral exchange rates, which are also useful for forecasting applications. In a seminal contribution, Meese and Rogoff (1983) provided some early evidence that exchange rates are difficult to predict, at least in the short run. Using a set of theoretical models in the spirit of Dornbusch (1976), Frankel (1979), and Hooper and Morton (1982), to guide the choice of covariates included in a forecasting regression, Meese and Rogoff (1983) found that a simple random walk benchmark is difficult to outperform for most major exchange rate pairs. One reason for the dismal performance of most empirical and structural models is that, within a standard asset pricing framework, the high persistence of the underlying fundamentals in light of a discount factor near unity translates into highly persistent exchange rates. As a consequence, a random walk appears to be a benchmark difficult to beat (see Engel & West, 2005).

Over the years, a plethora of alternative econometric techniques emerged that provide more sophisticated means for analyzing exchange rate data to successfully improve longer term predictions. The literature on unit roots and cointegration, for example, provided tools to explicitly discriminate between short‐term movements of a given currency pair and its long‐run behavior. Mark (1995), for instance, applied an error correction model to a set of four exchange rates against the US dollar. Within this error correction framework, the exchange rate is assumed to return to its long‐run equilibrium value determined by a simple monetary model, with short‐run fluctuations driven by lagged changes of the exchange rate and its fundamentals. The finding that exchange rates tend to be predictable in the medium and long run sparked a series of related contributions that corroborate this result for different periods and currency pairs (Groen, 2000; Mark & Sul, 2001; Rapach & Wohar, 2002).

More recently, several studies emphasized the usefulness of accounting for nonlinearities in the underlying econometric models to provide more precise exchange rate predictions (see, for example, Byrne, Korobilis, & Ribeiro, 2016; Canova, 1993; Huber, 2016, 2017; Huber & Zörner, 2019; Mark, 2009; Sarno, Valente, & Wohar, 2004). The majority of this literature deals with the question on whether a given empirical model, that is loosely based on an underlying structural model, outperforms a set of competing models. In this context, introducing nonlinearities boils down to allow for time‐varying error variances and/or time‐varying regression coefficients for a certain structural model.

However, another key source of nonlinearities could stem from the fact that the underlying theoretical model changes over time, potentially jeopardizing the predictive fit of the econometric specification. 1Recent contributions, dealing with this issue, are Wright (2008), Beckmann and Schüssler (2016), Beckmann, Koop, Korobilis, and Schüssler (2018), and Byrne, Korobilis, and Ribeiro (2018). For instance, the recent success of Taylor rule‐based models (see Engel & West, 2006; Molodtsova, Nikolsko‐Rzhevskyy, & Papell, 2008, 2011; Molodtsova & Papell, 2009) can be attributed to the fact that involved central banks adopted a policy rule closely related to a Taylor rule. With short‐term interest rates reaching the zero lower bound (ZLB) and central banks starting to implement unconventional monetary policy measures, the question arises whether a Taylor rule still proves to be an adequate exchange rate model. In fact, recent literature on nonlinear Taylor rules suggests that during the ZLB, Taylor rule‐based models loose their momentum against simple random walk specifications (Byrne et al., 2016; Huber, 2017).

In this paper, we contribute to the literature by acknowledging this empirical evidence and propose a modeling framework capable of handling model instability over time in a flexible manner. This is achieved by proposing a Markov‐switching (MS) regression model with each regime being characterized by different covariates arising from a set of structural exchange rate models. In contrast to the existing literature, which relies on dynamic Bayesian model averaging techniques, our approach is an integrated modeling device. In addition, the introduction of time‐varying transition probabilities allows assessment of how the likelihood of a given structural model changes over time, depending on selected early‐warning indicators. As signal variables, we adopt the (lagged) interest rates of the home country and the USA. This specification is motivated by the observation that Taylor rule fundamentals are good predictors in the period before the global financial crisis (with policy rates being significantly larger than zero), but are known for their weak performance in the aftermath of the crisis (characterized by policy rates close to zero).

We assess the merits of the proposed approach using a forecasting exercise for eight different exchange rates against the US dollar. By considering the resulting regime allocation and the transition probabilities, we examine whether structural models indeed tend to change and how this is related to movements in policy rates. The findings indicate that allowing for time‐varying probabilities is a key feature, pointing towards a strong relationship between policy rates and the underlying transition distribution of the Markov process. In terms of forecasting, we find that our proposed model improves upon the random walk for selected currencies, both in terms of point and density predictions. The improvements for point forecasts are, however, muted. Comparing different model features reveals that a model based on a larger set of fundamentals from various structural models is also competitive when combined with shrinkage priors and nonlinearities (in the form of MS).

The remainder of this paper is organized as follows. Section 2 discusses the four structural exchange rate models adopted, while Section 3 proposes the econometric framework. The empirical application is presented in Section 4. The final section summarizes and concludes the paper. A technical Appendix provides details on the estimation algorithm adopted.

## 2 THEORETICAL EXCHANGE RATE MODELS

In this section, we briefly discuss the main theoretical underpinnings to be used to guide covariate inclusion in the empirical model as well as to structurally identify the different regimes considered in our nonlinear regression framework.

The point of departure for the discussion is a set of macroeconomic and financial quantities stored in an *R*‐dimensional vector ***X***_*t*_:


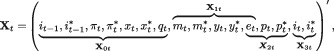
 with *i*_*t*−1_ denoting the lagged short‐term interest rate, *π*_*t*_ inflation, *x*_*t*_ output gap, *m*_*t*_ money supply, *y*_*t*_ income, *p*_*t*_ price level, while the real exchange rate is denoted by *q*_*t*_ and the nominal exchange rate by *e*_*t*_. 2Asterisks denote US quantities. Moreover, *y*_*t*_, *m*_*t*_, *p*_*t*_, *q*_*t*_ and *e*_*t*_ are measured in logarithms. For simplicity, we suppress subset‐specific intercepts. The subsets of ***X***_*t*_, ***X***_*jt*_ (*j*=0,…,3), represent covariates associated with the different structural models that we describe next.

### 2.1 A taxonomy of selected models of exchange rate determination

In the following, we provide a brief taxonomy of the theoretical models considered that determine the specific partitions of ***X***_*t*_.

- Our starting point is the model based on *Taylor rule fundamentals* (see, Molodtsova & Papell, 2009, for a recent forecasting study). This specification assumes that the set of predictors is given by ***X***_0*t*_ and thus includes the lagged short‐term interest rate, inflation and the output gap of both the home country and the USA, and the real exchange rate. This model has proved to be successful in terms of describing exchange rate movements, both in‐sample (Engel & West, 2006) and out‐of‐sample (Molodtsova et al., 2008, 2011). However, one critical assumption of this specification is that the central bank at home and in the USA is actively pursuing a Taylor rule‐based monetary policy strategy. Especially during the recent period at the ZLB, this assumption could be violated, effectively leading to an inferior model fit.
- The second model considered is the *long‐run monetary model*. The monetary model assumes that the covariates are given by ***X***_1*t*_ and include data on domestic and US money supply as well as cross‐country differences in income for a given income elasticity. As mentioned by Rapach and Wohar (2002), the long‐run monetary model simply states that the price level of the home country and the USA is determined by the money supply and the level of production. Assuming purchasing power parity (PPP) and uncovered interest rate parity (UIP), one is able to relate the change in the exchange rate to supply and demand for money.
- Third, we consider a model based on PPP. This model selects ***X***_2*t*_, leading to a regression model that includes domestic and US price indices. PPP originates from the law of one price, which in turn implies that the real exchange rate is supposed to revert to a long‐run equilibrium level determined by relative prices. If this turns out to be true, the real exchange rate is a stationary process. However, Sarno (2005) highlighted substantial persistence in real exchange rates. The convergence towards PPP is thus slow in the long run and, real exchange rates typically display pronounced deviations from their PPP‐implied fundamentals in the short run.
- Finally, we also augment our forecasting regression with the UIP model. By selecting ***X***_3*t*_, this model simply establishes a relationship between the change in the exchange rate and the interest rate differential between the home country and the USA. Following Chinn (2006), UIP implies a positive one‐to‐one relationship between the interest rate differential and changes in the exchange rate. A positive change in the interest differential may be potentially followed by an immediate and persistent appreciation in the short run, implying that UIP does not hold immediately. 3See, for instance, Chinn (2006) and Engel (2014) who observe coefficients that are less than one or even negative.

All these models have been shown to possess some merit in terms of predictive power. However, several recent studies find remarkable heterogeneity with respect to the fundamental model adopted (see, among others, Beckmann & Schüssler, 2016; Beckmann et al., 2018; Byrne et al., 2018; Wright, 2008).

## 3 CONTROLLING FOR MODEL INSTABILITY IN EMPIRICAL EXCHANGE RATE MODELS

In this section, we propose a model that controls for dynamic model instability by specifying a nonlinear econometric framework. After summarizing the model structure in Section 3.1, we highlight the prior setup adopted in Section 3.2.

### 3.1 An MS model specification

We now turn to describing the proposed MS model with time‐varying transition probabilities (MS‐TVP). The key feature of our proposed framework is that it allows for switching between the fundamentals implied by *K* competing theoretical exchange rate models. We assume that exchange rate returns Δ*e*_*t*_ follow an MS‐TVP model given by

(1) $$\Delta e_{t}=X_{S_{t}t-1}^{\prime }\beta_{S_{t}}+\eta_{t},$$

where *S*_*t*_ ∈ {0,…,*K*−1} follows a first‐order Markov process, ***β***_*k*_ represents a vector of dimension *M*_*k*_ that collects the state‐specific coefficients of state *S*_*t*_=*k*, while $\eta_{t}\;∼\;N(0,\sigma_{S_{t}}^{2})$ is a white noise shock with regime‐specific variance $\sigma_{S_{t}}^{2}$. Note that each ***β***_*k*_ may exhibit different dimensions. We depart from the traditional literature on MS models (see, among many others, Amisano & Fagan, 2013; Billio, Casarin, Ravazzolo, & Van Dijk, 2016; Casarin, Sartore, & Tronzano, 2018; Engel, 1994; Filardo, 1994; Hamilton, 1994; Huber & Fischer, 2018; Kaufmann, 2015) by assuming that the regimes are characterized by competing structural exchange rate models, implying that different fundamentals enter the predictive exchange rate regression at different points in time.

In the spirit of Belmonte & Koop, 2014 and Frühwirth‐Schnatter, 2006, we introduce a selection matrix $D_{S_{t}}$ that entails switching between *K* alternative model specifications:

(2) $$\Delta e_{t}=X_{t-1}^{\prime }D_{S_{t}}\beta +\eta_{t},$$

with ***X***_*t*−1_ denoting an *R*‐dimensional vector of the full set of economic fundamentals. We define $\beta ={\left(\beta_{0}^{\prime },\text{…},\beta_{K-1}^{\prime }\right)}^{\prime }$ as a stacked *M*‐dimensional vector of regime‐specific coefficients with $M=\sum_{j=0}^{K-1}M_{j}$, and $\sigma^{2}={\left(\sigma_{0}^{2},\text{…},\sigma_{K-1}^{2}\right)}^{\prime }$ collecting the *K* state‐specific variances.

The selection matrix ***D***_*k*_ of state *S*_*t*_=*k* is an *R*×*M*‐dimensional matrix with binary indicators that select ***β***_*k*_ and ***X***_*kt*−1_ while zeroing out the elements in ***β*** and ***X***_*t*−1_ associated with the remaining models. For instance, we effectively obtain the model based on Taylor rule fundamentals, characterized through *S*_*t*_=0, by setting

$$D_{0}=\left(\begin{array}{ccc} I_{7} & \text{…} & 0_{7\times 2}\\ \vdots & \ddots & \vdots \\ 0_{2\times 7} & \text{…} & 0_{2\times 2} \end{array}\right),$$

where **0**_*i*×*j*_ is an *i*×*j*‐dimensional matrix of zeros. Multiplying ***β*** from the left with ***D***_0_ yields

$$D_{0}\beta ={\left(\beta_{0}^{\prime },0_{5\times 1}^{\prime },0_{3\times 1}^{\prime },0_{2\times 1}^{\prime }\right)}^{\prime }.$$

From this discussion, it is clear that the matrix $D_{S_{t}}$ effectively controls the prevailing structural exchange rate model and the set of covariates to include in the state‐specific regression. Note that an MS kitchen‐sink regression is obtained by defining $D_{S_{t}}$ in such a way that in each state all economic indicators are included for all *t*.

### Time‐varying transition probabilities

Assuming constant transition probabilities is a standard (and potentially restrictive) assumption in MS models. Both Amisano and Fagan (2013) and Kaufmann (2015) propose treating the transition distributions as being dependent on additional covariates. Here, and since our model features *K* regimes, we follow Kaufmann and parametrize the transition probabilities by a multinomial logit specification. Given the forecasting evidence provided in the literature quoted above, we assume transition probabilities to depend on a measure of the monetary policy stance such as the policy rate. This captures the notion that if policy rates approach the ZLB, a Taylor rule‐based model might become inadequate and the likelihood of a regime shift could increase.

The multinomial likelihood reads

$$P(S_{t}=j|S_{t-1}=k,Z_{t},\gamma )=p_{kj,t}=\frac{\exp\left(Z_{t}^{\prime }\gamma_{kj}\right)}{1+\sum_{l=1}^{K-1}\exp\left(Z_{t}^{\prime }\gamma_{kl}\right)},$$

with ***γ***_*kj*_=(*γ*_0,*kj*_,…,*γ*_*N*−1,*kj*_)^*′*^ being category‐specific regression coefficients, collected in ***γ*** for all *k* and *j*=1,…,*K*−1. ***γ***_*kj*_ determines the sensitivity of the transition probability that drives the transition from the *k*th to the *j*th state. Moreover, ***Z***_*t*_ denotes an *N*‐dimensional vector of covariates, defined as

$$Z_{t}={\left(1,z_{t}^{\prime },ℐ[S_{t-1}=1],\text{…},ℐ[S_{t-1}=K-1]\right)}^{\prime },$$

where ***z***_*t*_ is a vector of early‐warning indicators that determine the dynamics of the transition probabilities, while ℐ(•) denotes an indicator function that equals one if its argument is true. This implies that we capture a first‐order Markov structure by including the previous states as additional regressors. Moreover, *γ*_0,*kj*_ represents the intercept of the reference state *S*_*t*−1_=0, and thus captures the corresponding time‐invariant state persistence. Consistent with Amisano and Fagan (2013), we let the coefficients associated with ***z***_*t*_ be regime invariant. It is worth noting that, if the coefficients of ***z***_*t*_ are zero, we obtain a classic fixed transition probability MS model.

The specific choice of ***z***_*t*_ proves to be an important modeling decision. As mentioned above, our goal is to include a measure of the (conventional) monetary policy stance to signal a potential transition from Taylor rule‐based policy making to discretionary monetary policy actions such as quantitative easing (QE). In our case, we assume two early‐warning indicators $z_{t}={\left({\overset{˜}{𝒾}}_{t-1},{\overset{˜}{𝒾}}_{t-1}^{*}\right)}^{\prime }$, the demeaned, lagged interest rate at home and in the USA. Demeaning covariates ensures that the time‐invariant part does not depend on the scale of ***z***_*t*_. A covariate can always be rewritten as a linear combination of its time‐varying component and its mean, with the latter determining the time‐invariant average state persistence (Kaufmann, 2015).

### 3.2 Prior specification and estimation strategy

Our approach is Bayesian, which implies that we have to carefully specify priors on the parameters of the model. Here, we follow George and McCulloch (1993, 1997) and specify a mixture of Gaussians prior on *β*_*ik*_, the *i*th element of ***β***_*k*_. The prior is centered on theoretically motivated restrictions in order to test whether these restrictions are consistent with the data. The prior mean is stored in an *M*_*k*_‐dimensional vector ${\underline{\beta }}_{k}$ and summarized in Table 1. We assume a symmetric Taylor rule with homogeneous coefficients for the home country and the USA and do not consider interest smoothing (see Molodtsova & Papell, 2009, for a detailed discussion). For the remaining models we center them on values consistent with the implied long‐run fundamental value.

**Table 1:** Prior mean ${\underline{\beta }}_{k}$ for each state

	Intercept	*i*_*t*−1_	$i_{t-1}^{*}$	*π*_*t*_	$\pi_{t}^{*}$	*x*_*t*_	$x_{t}^{*}$	*q*_*t*_	*m*_*t*_	$m_{t}^{*}$	*y*_*t*_	$y_{t}^{*}$	*e*_*t*_	*p*_*t*_	$p_{t}^{*}$	*i*_*t*_	$i_{t}^{*}$
${\underline{\beta }}_{0}$	0	0	0	1.5	−1.5	0.5	−0.5	0									
${\underline{\beta }}_{1}$	0								1	−1	1	−1	−1				
${\underline{\beta }}_{2}$	0												−1	1	−1		
${\underline{\beta }}_{3}$	0															1	−1

Formally, this prior reads

(3) $$\beta_{ik}|\delta_{ik}∼N\left({\underline{\beta }}_{ik},\tau_{ik,1}^{2}\right)\delta_{ik}+N\left({\underline{\beta }}_{ik},\tau_{ik,0}^{2}\right)(1-\delta_{ik}),$$

where we let $\tau_{ik,0}^{2}$ and $\tau_{ik,1}^{2}$ be prior variances (with $\tau_{ik,1}^{2}\gg \tau_{ik,0}^{2}$), for *i*=1,…*M*_*k*_, and ${\underline{\beta }}_{ik}$ denotes the *i*th element of ${\underline{\beta }}_{k}$. The first mixture component is referred to as the “slab” component, introducing almost no prior information, while the second is called the “spike” component, tightly centered around the prior mean ${\underset{-}{\beta }}_{ik}$. The indicator *δ*_*ik*_ serves to select the mixture component used. Following the semiautomatic approach of George, Sun, D., and Ni (2008) we scale the prior variances, $\tau_{ik,0}^{2}$ and $\tau_{ik,1}^{2}$, with the variances of the ordinary least square estimates of the underlying structural model in state *S*_*t*_=*k*.

This modeling approach constitutes a data‐driven way of assessing whether coefficients should be pushed towards theoretically motivated restrictions or allowed to be closely related to the corresponding maximum likelihood estimate. Thus, if *δ*_*ik*_=0, the posterior estimate of *β*_*ik*_ is strongly pushed towards the prior restriction ${\underline{\beta }}_{ik}$ while, in the opposite case, only little prior information on *β*_*ik*_ is introduced.

In what follows, we store all regime‐specific indicators in a vector $\delta_{k}={\left(\delta_{1k},\ldots ,\delta_{M_{k}}\right)}^{\prime }$ that corresponds to the block of ***β*** associated with the *k*th structural model. Each element of the latent variable ***δ***_*k*_ is a priori independently Bernoulli distributed:

$$\begin{array}{ccc} p(\delta_{ik}=1|S_{t}=k)= & {\underline{\omega }}_{ik}, & \\ p(\delta_{ik}=0|S_{t}=k)= & 1-{\underline{\omega }}_{ik}, \end{array}$$

for hyperparameters ${\underline{\omega }}_{ik}$ chosen by the researcher. A reasonable choice is ${\underline{\omega }}_{ik}=0.5$, for all *i*,*k*, implying an equal prior probability of introducing significant prior information or using a relatively loose prior. 4When considering a weakly informative coefficient prior, we define ${\underline{\omega }}_{ik}$ as being one for including all state‐specific coefficients with certainty.


For the variances ***σ*** ^2^, we assume an independent inverse Gamma prior for each element $\sigma_{i}^{2}\;(i=0,\ldots ,K-1)$. More specifically, we set

$$\sigma_{i}^{2}∼G^{-1}(a_{0},A_{0}),$$

with *a*_0_ and *A*_0_ being scalars. The specific values for *a*_0_ and *A*_0_ are chosen to be weakly informative with hyperparameters *a*_0_=0.01, *A*_0_=0.01.

The prior distribution on the initial state is set to *p*(*S*_0_=*k*)=1/*K*, for all *k* (Kaufmann, 2015). Finally, for the coefficients of the multinomial logit model, we adopt a weakly informative and symmetric prior across all states. That is,

$$\gamma_{kj}∼N(0,\underline{V}),$$

for all *k* and *j*=1, …, *K*−1 with $\underline{V}=\zeta I_{N}$, and *ζ* denoting a scalar. In the empirical application we set *ζ*=100.

In a Bayesian framework, we combine the likelihood with the prior to obtain the posterior distribution. In our case, the joint posterior density is intractable. Fortunately, the full conditional posterior distributions take simple forms, permitting Gibbs updating steps. The Markov chain Monte Carlo (MCMC) algorithm is described in more detail in the Appendix. In the empirical application, we repeat the algorithm 80,000 times, discard the first 30,000 draws as burn‐in and define a thinning factor of 10, thus basing inference on 5,000 draws from the joint posterior.

Before proceeding to the empirical application, a brief word on identification is in order. Identification is necessary for structural interpretation of the states, but is not relevant if interest centers exclusively on the predictive density of the model (Frühwirth‐Schnatter, 2001, 2006). 5MS models might suffer from identification problems due to the invariance of the likelihood with respect to permutations of the *K*! possible labeling of the regimes, resulting in *K*! modes. Recall that in the present model each regime is characterized by a different set of fundamentals, reflecting different theoretical exchange rate models. By exploiting the specific structure of the theoretical models, we impose inequality constraints on the coefficients by selecting the fundamentals for each regime. The only potential source of nonidentifiability occurs in the case of more than one state pointing towards a random walk. However, pushing coefficients in the direction of theoretical guided values is sufficient to disentangle regimes and fully identify the model. When considering the alternative specification, in which we always include all predictors, identification is certainly an issue. Moreover, each state is implicitly centered on a random walk a priori. In this case, we apply a permutation sampling step and solely focus on predictive densities.

## 4 EMPIRICAL APPLICATION

This section starts by briefly describing the data set and forecasting design adopted in Section 4.1. We then discuss key in‐sample features of the model in Section 4.2. Finally, Section 4.3 presents the main forecasting results, discussing the point and density forecasting performance of all models considered.

### 4.1 Data, forecasting design, and competing models

In this paper, our aim is to forecast bilateral exchange rates for Australia, Canada, Japan, Norway, South Korea, Sweden, Switzerland and the UK relative to the US dollar. We collect monthly data on nominal exchange rates, industrial production, monetary aggregates, 3‐month money market rates and consumer price indices for all countries under consideration (see Tables 2 and 3 for details).

**Table 2:** Transformation of variables

Variable	Description	Transformation
EXR	Nominal exchange rate	log difference
IP	Industrial production	log
M	Money aggregate	log
3M‐IR	3M Money market rate	—
CPI	Consumer price index	log
INF	Inflation	log differences of CPI
REXR	Real exchange rate	log(EXR)+log(CPI*)−log(CPI)
IP‐GAP^a^	Output gap	HP filter

a *λ*=14,400 for monthly data.

**Table 3:** Sources of economic fundamentals

Country	Coverage	EXR	IP (2010 = 100)	M	3M‐IR	CPI (2010 = 100)
Australia (AU)	1975:M06–2017:M09	IFS	OECD	M1, OECD	OECD	OECD
Canada (CA)	1973:M01–2017:M09	IFS	OECD	M1, OECD	OECD	OECD
Japan (JP)	1973:M01–2017:M03	IFS	OECD	M1, OECD	IFS	OECD
Norway (NO)	1979:M01–2017:M09	IFS	OECD	M1, OECD	OECD	OECD
South Korea (KR)	1981:M01–2017:M09	IFS	IFS	M1, OECD	IFS	OECD
Sweden (SE)	1973:M01–2017:M06	IFS	OECD	M3, OECD	IFS	OECD
Switzerland (CH)	1974:M01–2017:M09	IFS	OECD	M1, OECD	OECD	OECD
United Kingdom (UK)	1973:M01–2017:M02	IFS	OECD	M0, IMF/FRED	IFS	OECD
United States (USA)	1973:M01–2017:M09		OECD	M1, OECD	OECD	OECD

*Note.* All quantities are seasonally adjusted, except EXR and 3M‐IR. IP of Australia and Switzerland are interpolated to monthly frequency with cubic spline.

In order to assess whether time‐varying transition probabilities improve predictive accuracy, the proposed model framework is benchmarked with MS specifications featuring fixed transition probabilities (labeled MS‐FT), as well as standard structural exchange rate models that are estimated under weakly informative priors (labeled linear). These linear benchmarks are based on Taylor rule, monetary, PPP, and UIP fundamentals. The set of competing models can thus be divided into three overall classes: MS‐TVP, MS‐FT, and linear. Furthermore, we consider not only theoretically motivated MS‐TVP and MS‐FT specifications, but also models that include all macroeconomic indicators of ***X***_*t*−1_ within each state (labeled kitchen‐sink). For the kitchen‐sink regressions, we consider different numbers of states, ranging from two to four regimes. Moreover, to allow for state‐specific shrinkage in kitchen‐sink regressions, the SSVS prior described above is centered on zero and different state‐specific indicators are estimated. To assess the role of allowing for heteroskedasticity in forecasting exchange rates, we also consider MS‐TVP specifications with state‐specific variances. All models are then benchmarked to the random walk without drift.

We evaluate predictive accuracy by means of a recursive pseudo out‐of‐sample forecasting exercise. This implies choosing an initial estimation period that ranges from *t*=1 up to *t*=*T*_0_, with the remaining periods used as a hold‐out sample. In the present application, we estimate all models using data up to 2004:M12 and then proceed by computing *h*‐step‐ahead predictions for *t*=*T*_0_+1. After obtaining draws from the corresponding predictive distributions, we consequently expand an initial estimation period by 1 month. This procedure is repeated until the end of the sample is reached.

To rank forecasts, we rely on cumulative squared forecast errors (CSFEs) to assess the quality of point forecasts. As point predictions, we take the posterior median of the predictive density. Turning to density forecasts, we follow Geweke and Amisano (2010) and rely on the log predictive score (LPS) to measure density forecasting accuracy. This has the advantage that, conditional on the proposed model and data, uncertainty surrounding the parameters and latent quantities is integrated out. After obtaining the LPS, we compute log predictive Bayes factors (LBFs) for the entire hold‐out sample by computing the difference between the LPS of a given model and the LPS of the random walk.

### 4.2 Some evidence for model instability

**Figure 1:**
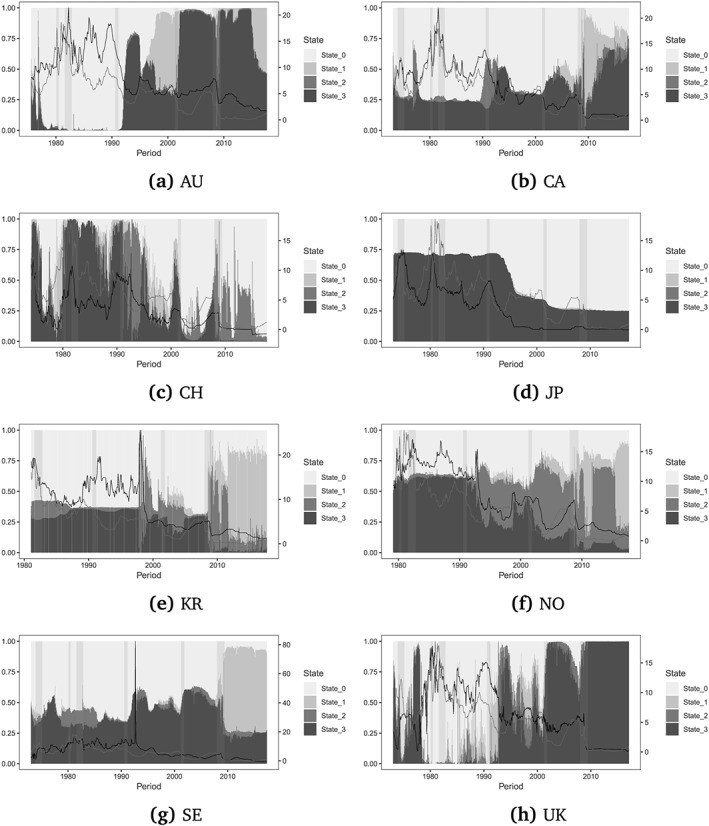
Filtered average posterior state probabilities for an SSVS prior and a common variance across states. State 0 indicates Taylor rule fundamentals, state 1 monetary fundamentals, state 2 PPP fundamentals, and state 3 UIP fundamentals. The vertical bars (yellow) indicate NBER recessions for the USA. The black solid line depicts the country‐specific interest rate and the red solid line the interest rate for the USA. The left‐axis shows the stacked probabilities and the right‐scale the values of interest rates [Colour figure can be viewed at http://wileyonlinelibrary.com]

In this subsection we assess whether our proposed model signals significant shifts in the underlying structural representation. Figure 1 summarizes the mean of the filtered state probabilities for the eight exchange rates considered. In general, we observe that the regime dynamics across countries share one common feature. The models based on Taylor rule (state 0) and the UIP fundamentals (state 3) appear to dominate before the global financial crisis in 2008/2009. After that period, however, model evidence changes significantly for the majority of countries. More precisely, models based on monetary (state 1) and PPP (state 2) fundamentals tend to receive more posterior support.

Compared to the remaining currencies, the Swiss franc (see Figure 1c) exhibits a somewhat higher regime‐switching frequency. In addition, Figure 1 suggests that hitting the ZLB does shift filtered probabilities, pointing to regimes other than Taylor rule fundamentals (state 0) for countries such as Australia, Canada, South Korea, Sweden, and the UK. 6Note that Australia never hit the ZLB during the sample, but the USA did. Moreover, it is worth emphasizing that Japan already hit the ZLB in the midst of the 1990s. Countries such as Japan and Switzerland, on the other hand, indicate an opposite dynamic, namely a shift of probabilities towards the Taylor rule state.

Taking a closer look at the UK, Taylor rule fundamentals are the predominant regime, reflecting the fact that these quantities tend to describe exchange rates well in times when the primary policy rule of the Bank of England is the Taylor rule. After this period, transition probabilities change during the crisis of the European Monetary System. After the financial crisis, and upon hitting the ZLB, the model based on Taylor rule fundamentals receives only limited posterior support. It is noteworthy that after 2010 the short‐term interest rate (both at home and in the USA) is stuck at zero (and almost constant). This implies that the model based on interest fundamentals closely mimic a random walk during this period, even without introducing shrinkage.

**Figure 2:**
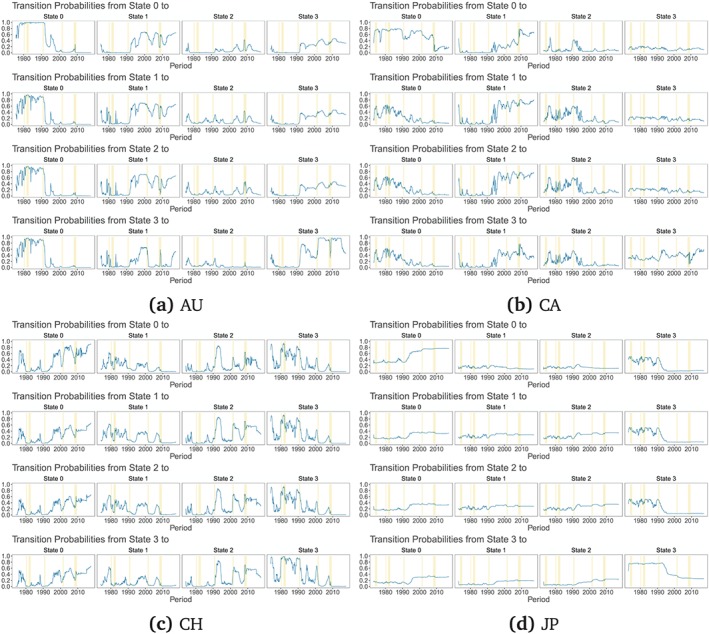
The blue line depicts the posterior mean of time‐varying transition probabilities for each state with an SSVS prior and a common variance across states. State 0 indicates Taylor rule fundamentals, state 1 monetary fundamentals, state 2 PPP fundamentals, and state 3 interest rate fundamentals. The vertical bars (yellow) indicate NBER recessions for the USA [Colour figure can be viewed at http://wileyonlinelibrary.com]

**Figure 3:**
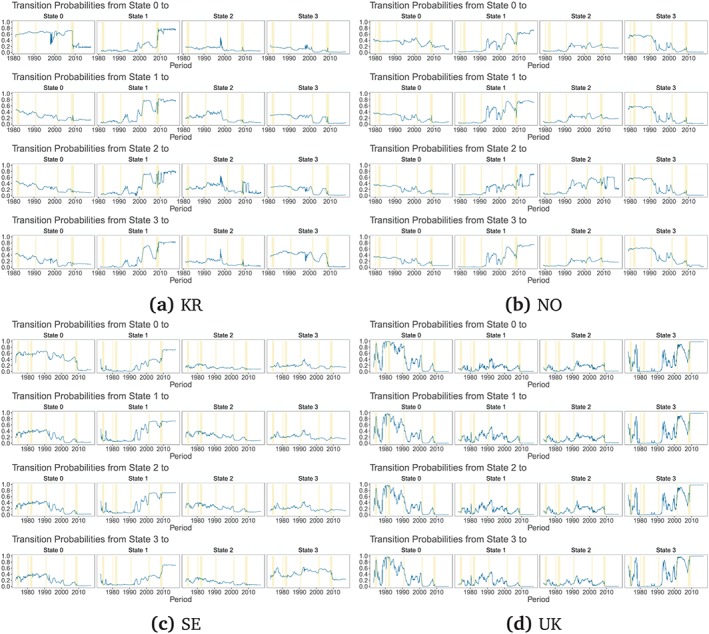
The blue line depicts the posterior mean of time‐varying transition probabilities for each state with an SSVS prior and a common variance across states. State 0 indicates Taylor rule fundamentals, state 1 monetary fundamentals, state 2 PPP fundamentals, and state 3 interest rate fundamentals. The vertical bars (yellow) indicate NBER recessions for the USA [Colour figure can be viewed at http://wileyonlinelibrary.com]

The transition probabilities, depicted in Figures 2 and 3, generally track the movements in filtered state probabilities, providing considerable evidence of time‐varying transition distributions. Our findings thus suggest that a measure of the monetary policy stance at home and in the USA tends to drive transitions between structural models. This is consistent with our conjecture that during the period of the ZLB using Taylor rule‐based exchange rate models might be inappropriate, at least from an in‐sample perspective.

### 4.3 Forecasting results

In this section, interest centers on the predictive performance of our proposed MS‐TVP specification. The discussion in the last section provided evidence in favor of time‐varying transition probabilities for several exchange rate pairs. This suggests that parametrizing the transition distributions with additional covariates helps to avoid situations where the model gets stuck within a certain state. Amisano and Fagan (2013) and Kaufmann (2015) highlight this issue and point towards advantages of explaining the regime‐switching behavior of the model as opposed to a model based on constant transition probabilities. However, the key question is whether this additional flexibility also improves predictive performance. We answer this question using both point and density forecasts.

#### 4.3.1 Point forecasts

**Figure 4:**
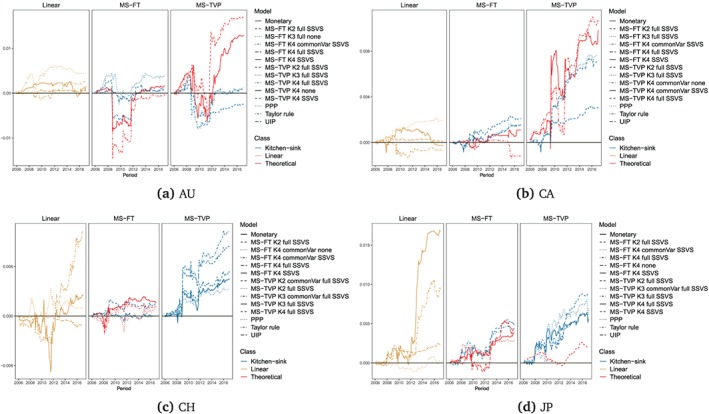
Difference to the random walk of one‐step‐ahead CSFEs for the full hold‐out sample for Australia, Canada, Switzerland, and Japan. “Linear” specifies the linear univariate exchange rate regressions. For the Markov‐switching models with time‐varying transition probabilities (MS‐TVP) and models with fixed transition probabilities (MS‐FT), *K*[2–4] specifies the number of states. We evaluate all models with a common state variance (commonVar) and individual state variances, with both the theoretical state and the kitchen‐sink (full) state specification. Moreover, we estimate all Markov‐switching models with and without an SSVS prior. We consider the five best‐performing MS‐TVP and and five best MS‐FT models according to the CSFEs at the end of the hold‐out sample [Colour figure can be viewed at http://wileyonlinelibrary.com]

**Figure 5:**
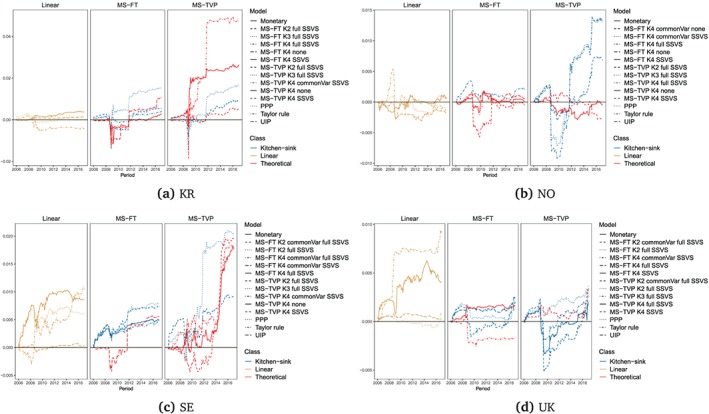
Difference to the random walk of one‐step‐ahead CSFEs for the full hold‐out sample for South Korea, Norway, Sweden, and the UK. “Linear” specifies the linear univariate exchange rate regressions. For the Markov‐switching model with time‐varying transition probabilities (MS‐TVP) and models with fixed transition probabilities (MS‐FT), *K*[2–4] specifies the number of states. We evaluate all models with a common state variance (commonVar) and individual state variances, with both the theoretical state and the kitchen‐sink (full) state specification. Moreover, we estimate all Markov‐switching models with and without an SSVS prior. We consider the five best‐performing MS‐TVP and and five best MS‐FT models according to the CSFEs at the end of the hold‐out sample [Colour figure can be viewed at http://wileyonlinelibrary.com]

Figures 4 and 5 present the evolution of CSFEs of one‐step‐ahead forecasts for the best‐performing models across the set of model classes. We consider all linear predictive exchange rate regressions and the five best‐performing MS‐TVP and MS‐FT models, according to the CSFEs at the end of the hold‐out sample. The CSFEs of all models are shown as a difference between them and the CSFEs of the random walk benchmark. Thus values below zero indicate more accurate point forecasts than random walk predictions. Here, we focus on one‐step‐ahead forecasts, since we find that models that perform well at the one‐step‐ahead horizon also do well for *h*>1 periods ahead. 7Additional results for *h*(>1)‐step‐ahead forecasts are provided upon request. When considering density forecasts, we report the results for higher order predictions as well.

Turning to the actual results, we observe pronounced differences across countries. For instance, in Australia, Canada, Norway, Sweden, and the UK, modeling nonlinearities pays off, in particular during periods of financial turmoil, outperforming forecasts of linear models and the random walk benchmark. Therefore, one interesting finding is that controlling for heteroskedasticity also tends to exert a positive effect on the point forecasting performance during volatile periods of the business cycle.

By contrast, for South Korea and Switzerland, the random walk appears to be hard to beat. In general, we observe CSFE differences that are close to zero when averaged over the full hold‐out sample. This indicates that including more information does not necessarily translate into improved point predictions relative to a simple no‐change forecast for these two economies. Again, we find some heterogeneity in relative forecasting performance over time.

**Figure 6:**
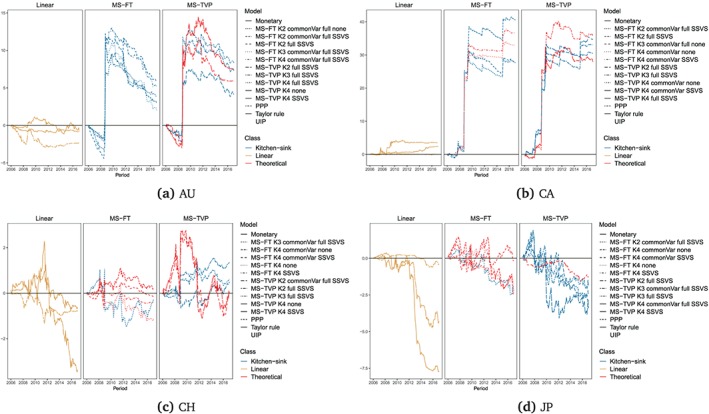
Cumulative one‐step‐ahead LBFs (random walk benchmark) for the full hold‐out sample for Australia, Canada, Switzerland, and Japan. “Linear” specifies the linear univariate exchange rate regressions. For the Markov‐switching models with time‐varying transition probabilities (MS‐TVP) and models with fixed transition probabilities (MS‐FT), *K*[2–4] specifies the number of states. We evaluate all models with a common state variance (commonVar) and individual state variances, with both the theoretical state and the kitchen‐sink (full) state specification. Moreover, we estimate all Markov‐switching models with and without an SSVS prior. We consider the five best‐performing MS‐TVP and and five best MS‐FT models according to cumulative LBFs at the end of the hold‐out [Colour figure can be viewed at http://wileyonlinelibrary.com]

**Figure 7:**
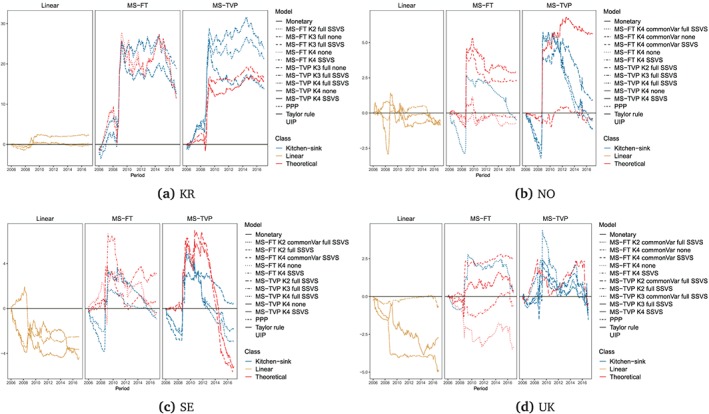
Cumulative one‐step‐ahead LBFs (random walk benchmark) for the full hold‐out sample for South Korea, Norway, Sweden, and the UK. “Linear” specifies the linear univariate exchange rate regressions. For the Markov‐switching models with time‐varying transition probabilities (MS‐TVP) and models with fixed transition probabilities (MS‐FT), *K*[2–4] specifies the number of states. We evaluate all models with a common state variance (commonVar) and individual state variances, with both the theoretical state and the kitchen‐sink (full) state specification. Moreover, we estimate all Markov‐switching models with and without an SSVS prior. We consider the five best‐performing MS‐TVP and and five best MS‐FT models' cumulative LBFs at the end of the hold‐out [Colour figure can be viewed at http://wileyonlinelibrary.com]

Turning to the performance of the theoretically inspired MS‐TVP specifications, we observe strong forecasting accuracy for Japan, Norway, South Korea, and the UK, at least for one specification (marked in red in Figures 6 and 7). On the other hand, it appears that kitchen‐sink specifications dominate theoretically inspired regimes for Australia, Canada, Switzerland, and Sweden. In particular, this holds true for Switzerland as, indicated by the absence of a red‐colored line in Figure 6c for MS‐TVPs.

When comparing MS‐TVP to MS‐FT models, we observe that MS‐FT specifications appear to be more robust over time in terms of CSFEs. This can be seen by noting that the forecast errors of the MS‐FTs feature fewer outliers. In general, we find a better performance of MS‐TVP models for Australia, Japan, and Norway, at least for one model, compared to MS‐FT models, which display a remarkable performance for the UK and Canada.

Accuracy differences across simple linear specifications appear to be diverse. For the UK, Sweden, and Japan, we observe an inferior predictive performance for at least two linear models. In particular, models based on monetary fundamentals exhibit a weak forecast performance relative to the remaining models under scrutiny. For Australia, Canada, and South Korea, all linear models do well and show a similar point forecast performance as the random walk. When focusing on Taylor rule fundamentals, the linear regression performs well at the beginning of the hold‐out sample (see, e.g., the results for the UK), but exhibits a systematic accuracy loss in periods after the financial crisis. This can be explained by the fact that Tayor rule‐based models build on the assumption that both central banks' monetary policy might be well described by a Taylor rule. However, after the financial crisis, interest rates hit the ZLB and central banks increasingly adopted nonstandard policy measures. This, in turn, leads to a deteriorating performance of this model class, effectively confirming findings reported in the recent literature (see, e.g., Byrne et al., 2016; Molodtsova & Papell, 2012).

#### 4.3.2 Density forecasts

Tables 4 and 5 depict a summary of all models' LBFs for all currency pairs considered. Values larger than zero point towards outperformance of a model relative to the random walk, while negative values signal a weaker predictive performance when benchmarked against the random walk. To provide a dynamic picture of LBFs over time, Figures 6 and 7 show the LBFs of all linear predictive exchange rate regressions and the five best‐performing MS‐TVP and MS‐FT models.

**Table 4:** Cumulative one‐, three‐, and 12‐step‐ahead LBFs (random walk benchmark) at the end of the full hold‐out sample summarized for Australia, Canada, Switzerland, and Japan. Values highlighted green (red) are larger (smaller) than zero, indicating a better (weaker) performance compared to the random walk. Best model in bold [Colour table can be viewed at http://wileyonlinelibrary.com]

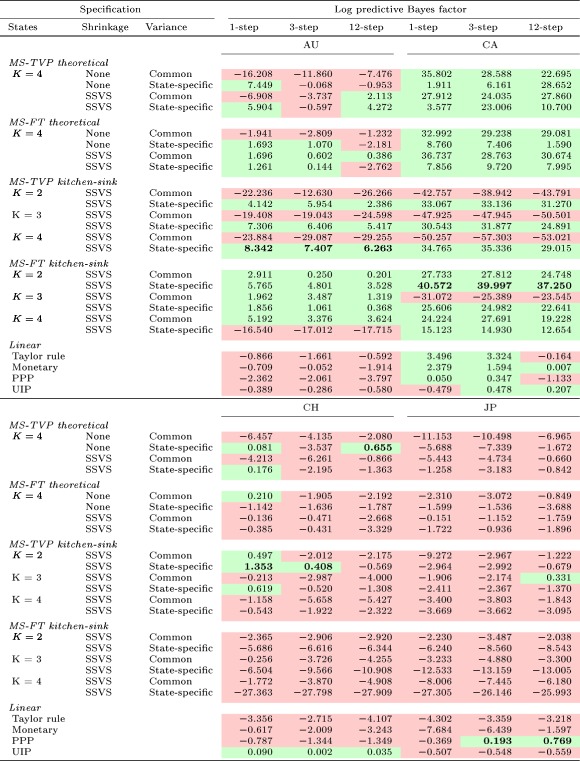

**Table 5:** Cumulative one‐, three‐, and 12‐step‐ahead LBFs (random walk benchmark) at the end of the full hold‐out sample summarized for South Korea, Norway, Sweden, and the UK. Values highlighted green (red) are larger (smaller) than zero, indicating a better (weaker) performance compared to the random walk. Best model in bold [Colour table can be viewed at http://wileyonlinelibrary.com]

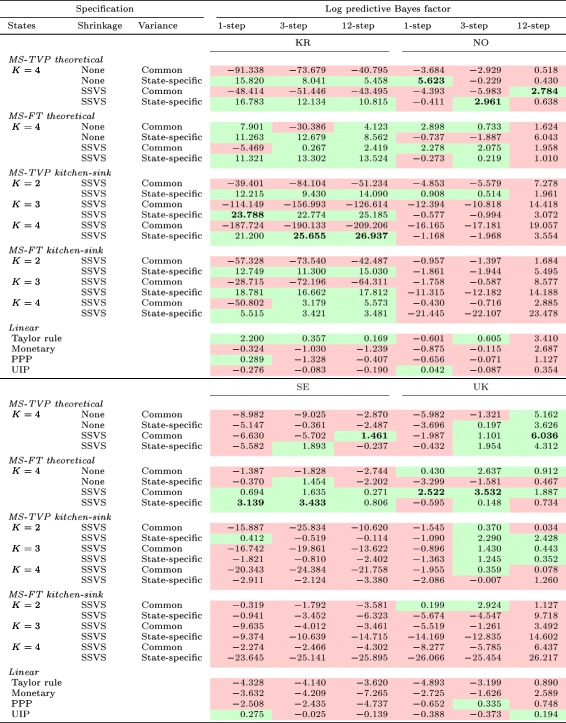

In general, both tables attest to nonlinear specifications' good predictive power, while linear models display a somewhat weaker forecast performance. Furthermore, our results suggest that nonlinear models that perform well in terms of point predictions also exhibit high predictive capabilities in terms of density forecasts. However, we find that predictive performance evolves differently for CSFEs and the LBFs. Density forecasts strengthen the argument in favor of nonlinear models, as the performance gains of the MS models are more sizable in periods of high exchange rate volatility, whereas accuracy losses in tranquil periods are rather muted.

Although we do not observe a single dominant nonlinear model across forecast horizons and countries, Tables 4 and 5 suggest that at least one nonlinear specification outperforms the random walk and the linear competitors. One exception proves to be Japan, for which the best specification is either the random walk (for one‐step‐ahead predictions) or the linear PPP model (for longer horizons).

For all forecast horizons considered, nonlinear specifications perform well for Australia, Canada, South Korea, Sweden, and the UK. Specifically, the MS‐TVP kitchen‐sink specification with four states coupled with state‐specific variances, constituting the most flexible specification, has good predictive power for Australia, Canada, and South Korea. For Australia, this model is the single best‐performing model across forecast horizons and, for South Korea, it is the best for three‐ and 12‐step‐ahead forecasts while being the second‐best specification in terms of one‐step‐ahead forecasts. Moreover, as shown in Figures 6 and 7, the poor performance of linear exchange rate models is even more pronounced for density forecasts. In particular, linear structural regressions display a sharp decline in predictive power after the financial crisis, corroborating findings in Molodtsova and Papell (2012) and Byrne et al. (2016).

Turning to the question of whether allowing for heteroskedasticity pays off in terms of density forecasting, we find substantial evidence that this additional flexibility proves to be important. The gains in predictive accuracy of nonlinear models can mainly be attributed to the more flexible variance specification of MS models. This can also be seen by comparing MS specifications with a common variance across states with MS models that feature state‐specific variances. For these models, we observe a slight accuracy premium relative to their homoskedastic counterparts. Allowing for state‐specific variances thus appears to be an important ingredient of a successful forecasting model. However, this increased flexibility comes at a cost. Specifically, we observe that during normal periods relative predictive accuracy declines steadily for several MS models (see, e.g., 7c). This observation confirms recent evidence provided in Abbate and Marcellino (2018).

Contrasting MS‐TVP with MS‐FT models, Figures 6 and 7 show that MS‐TVP models yield more precise density forecasts for Australia, Norway, South Korea, and Switzerland, while yielding an almost equivalent performance for Canada. Moreover, with time‐varying transition probabilities, theoretically motivated specifications play a more important role than for MS‐FT models. This points towards potential accuracy premia obtained by allowing for time‐varying transition probabilities and thus more precise inference surrounding the regime allocation.

Theoretically motivated MS‐TVP specifications exhibit good forecast performance for Australia, Canada, and Norway. Considering the results for Sweden, theoretically motivated MS‐TVPs perform well during prolonged periods of high exchange rate volatility. In periods of low volatility, however, this specification is slightly outperformed by competing specifications. By contrast, we find that for South Korea MS‐TVP kitchen‐sink regressions improve upon our proposed MS‐TVP models that allow for switching across structural exchange rate regressions. For the remaining countries in Figures 6 and 7, no clear pattern emerges when comparing both model types. For example, using structural MS‐TVP specifications yields strong increases in predictive power during the financial crisis but a weaker performance afterwards. For kitchen‐sink MS‐TVP regressions, we find no gain during the crisis but also no subsequent loss in the aftermath of the crisis.

Finally, we assess whether using shrinkage priors on the coefficients improves forecasts. Tables 4 and 5 indicate that shrinkage generally translates into better results in pairwise comparisons with the corresponding nonshrinkage counterpart. This observation is not consistent across models, countries, and forecast horizons considered. In particular, using a kitchen‐sink regression without shrinkage leads to poor forecast performance, as already shown by Wright (2008) and Li, Tsiakas, and Wang (2015). Turning to theoretically motivated MS models provides limited evidence on the usefulness of shrinkage priors. We conjecture that this stems from the fact that adopting MS specifications with theoretically defined regimes already introduces a certain amount of regularization that helps avoid overfitting.

## 5 CONCLUDING REMARKS

In this paper, we propose a Bayesian nonlinear time series model to forecast exchange rates. Our framework allows for dynamically switching between selected theoretical exchange rate models that are used to guide the specific choice of covariates included. As an additional novelty, we assume that the transition probabilities vary over time and depend on a measure of the monetary policy stance at home and in the USA. This feature enables us to capture breaks in the policy rule of the central bank that, in turn, could impact the prevailing structural exchange rate model adopted. For instance, our framework entails dynamically switching between models if short‐term interest rates hit the zero lower bound.

We use this framework to predict eight exchange rates vis‐à‐vis the US dollar. Considering the transition probabilities, we find considerable evidence of time variation. The filtered probabilities indicate that, especially after interest rates approach zero, model evidence shifts in favor of models other than the Taylor rule‐based models, highlighting the necessity to control for model uncertainty. To assess whether this feature also translates into predictive accuracy gains, we conduct a forecasting exercise. We find that results appear to be rather mixed, with point forecasts being only slightly better than those obtained from standard models. For density predictions, by contrast, we observe pronounced accuracy increases for selected exchange rates.

## Data Availability

The data sets as well as the estimation codes are available from the corresponding author upon request.

## APPENDIX A MCMC ALGORITHM

### A.1

Let ${\tilde{S}}_{T}={\left(S_{1},\text{…},S_{T}\right)}^{\prime }$ denote the full history of states and ${\tilde{Z}}_{T}$ be a *T*×*N*‐dimensional matrix of stacked covariates with the *t*th row given by ***Z***_*t*_′. After specifying appropriate starting values, the Gibbs sampler iterates through the following steps:

1 Sample parameters of the measurement equation ***θ***=(***β***,***σ*** ^2^,***δ***) from $p(\theta |{\tilde{S}}_{T},{\Delta \tilde{e}}_{T})$, with ${\Delta \tilde{e}}_{T}=(\Delta e_{1},\text{…}\Delta e_{T})$.
  - Conditional on the exchange rate data ${\tilde{e}}_{T}$ and allocation of the states ${\tilde{S}}_{T}$, sampling ***β*** and ***σ*** ^2^ can be done in a standard way by drawing the coefficients ***β***_*k*_ for *k*=0,…,*K*−1 in a block from a multivariate Normal distribution and the variances independently for each state from an inverse Gamma distribution.
  - Conditional on ***β***_*k*_ of state *S*_*t*_=*k*, one is able to sample the elements of ***δ***_*k*_ from a Bernoulli conditional posterior distribution.
2 For sampling the unknown states $p({\tilde{S}}_{T}|{\Delta \tilde{e}}_{T},\theta ,{\tilde{Z}}_{T},\gamma )$ we adapt the filtering algorithm put forth by Kim and Nelson (1999).
3 Following Polson, Scott, and Windle (2013), we sample multinomial coefficients from $p(\gamma |{\tilde{S}}_{T},{\tilde{Z}}_{T})$ to construct the time‐varying transition probabilities.
4 In case of label switching, we implement an additional permutation step outlined in Frühwirth‐Schnatter (2001) ensuring an equal probability of each mode appearing in the posterior distribution.

For the second step, we follow Kim and Nelson (1999) and sample ${\tilde{S}}_{T}$ in a block using a multimove Gibbs sampler. This implies simulating ${\tilde{S}}_{T}$ in block from the following joint conditional distribution:

$$p({\tilde{S}}_{T}|{\Delta \tilde{e}}_{T},\theta ,{\tilde{Z}}_{T},\gamma ),$$

and applying a forward filtering and backward smoothing algorithm (Frühwirth‐Schnatter, 2006).

Unfortunately, for step 3, there is no closed form of the multinomial logit posterior distribution. Frühwirth‐Schnatter and Frühwirth (2010) therefore introduced two auxiliary layers for estimating state‐specific utilities. Polson et al. (2013) took a different approach by representing the likelihood of multinomial logit model as a scale mixture of Gaussians with a Pólya Gamma mixing distribution. In a hierarchical form (by introducing a single layer of latent variables) this strategy implies that the multinomial coefficients are drawn from a set of Gaussians, and the auxiliary variables are sampled from a Pólya Gamma distribution. This approach has the advantage of fast convergence, simple implementation, and no need for an additional layer for approximating the error distribution.

Following Polson et al. (2013), conditional on the latent states ${\tilde{S}}_{T}$ and the auxiliary variables *ψ*_*jt*_ for *t*=1,…,*T* sampled from a $PG(\psi_{jt}|1,0)$ distribution, the posterior quantities are given by

$$\begin{array}{ccc} {\bar{V}}_{j}= & {\left({\tilde{Z}}_{T}^{\prime }\Psi_{j}{\tilde{Z}}_{T}+{\underline{V}}^{-1}\right)}^{-1} & \\ {\bar{\gamma }}_{kj}= & {\bar{V}}_{j}\left({\tilde{Z}}_{T}^{\prime }{\tilde{\kappa }}_{j}\right) \end{array}$$

where ***γ***_*k*0_ is zero for *k*=1,…,*K*−1 for reasons of identification, **Ψ**_*j*_=diag(*ψ*_*j*1_,…,*ψ*_*jT*_), and

$${\tilde{\kappa }}_{j}=\left(ℐ[S_{t}=j]-0.5\right)-\Psi_{j}Ce_{j}.$$

***C*** is a *T*×*K*−1‐dimensional matrix, with elements being defined as

$$C_{tl}=\log\left(\sum_{k\neq l}\exp(Z_{t}^{\prime }\gamma_{kj})\right),$$

and ***e***_*j*_ represents the unit vector with a one at the *j*th position.
